# Family Complexity and Parents’ Migration: The Role of Repartnering and Distance to Non-Resident Children

**DOI:** 10.1007/s10680-021-09594-0

**Published:** 2021-10-06

**Authors:** Roselinde van der Wiel, Niels Kooiman, Clara H. Mulder

**Affiliations:** 1 Faculty of Spatial Sciences, Population Research Centre, University of Groningen, Groningen, The Netherlands; 2 Statistics Netherlands, The Hague, The Netherlands

**Keywords:** Internal migration, Separation, Parents, Child residence, Repartnering, Family complexity

## Abstract

Recent research suggests that the increasing complexity of family life could be a factor in declines in internal migration (long-distance moves within countries). As many separated parents continue to share childcare responsibilities or have visiting arrangements, their mobility is naturally constrained. However, the relationship between family complexity and individual migration behaviour has never been studied explicitly. We compare separated parents with parents in two-parent families in their likelihood of migrating within the Netherlands. We use detailed records of parents’ partnership status and children’s residential situation. An event-history analysis was performed using register-based population data (*N* = 442,412). We find that separated, single parents are more likely to migrate than those in two-parent families. The same is true for repartnered mothers, while repartnered fathers are about as likely to migrate as fathers in two-parent families. Separated parents’ migration behaviour depends on where their children live. Having non-resident children who live some distance away is associated with a much higher likelihood of migrating than having resident children or non-resident children who live nearby. Having both resident and non-resident children who live nearby—shared residence (i.e. joint physical custody) is likely common in this situation—is associated with a considerably lower likelihood of migrating than having resident children only. Based on our findings, one would expect family complexities stemming from parental separation to be associated with higher rather than lower levels of migration. However, potential future increases in the number of parents who share physical custody after separation might lead to lower migration levels.

## Introduction

In recent years, it has been suggested that the increasing complexity of family life may be a factor in the reported declines in internal migration, i.e. long-distance moves within national borders^1^ (Cooke et al., 2016; Thomas et al., 2017a); such declines have been observed around the globe (Bell et al., 2018). Indeed, there is no doubt that family complexities, and the events producing these complexities, have consequences for spatial mobility.^2^ Separating and repartnering almost always requires one or both partners to move. For separated, single parents (denoted as ‘single parents’^3^ from here onwards) as well as for repartnered parents, local ties to non-resident and part-time resident children are likely to form a reason to prefer to stay in place rather than move elsewhere. Additionally, the locational needs and preferences of any resident children and a potential new partner complicate locational decisions. So, while the events of separating and repartnering increase mobility over both short distances (i.e. residential mobility) and long distances (i.e. internal migration), the resulting family complexities could restrict mobility over long distances.

The consequences of family complexity for internal migration are relevant to both societies and individuals. On the societal level, migration is regarded as a prerequisite for an efficient housing and labour market (e.g. Haas & Osland, 2014; Hensen et al., 2009). For separated individuals in particular, migration can promote recovery and well-being after separation by creating opportunities for repartnering, occupational progression, living closer to family (e.g. Albertini et al., 2018; Das et al., 2017) or seeking distance from the ex-partner (Duggan, 2007). On the downside, migration in a post-separation context will likely entail a disruption in the linked lives of separated parents and their children.

The literature has addressed several important dimensions of family complexity in relation to spatial mobility. A number of studies report on the elevated propensities of both residential mobility and internal migration following separation in various western countries (e.g. Clark & Huang, 2004; Clark & Davies Withers, 2007; Clark, 2013; Feijten & van Ham, 2007; Feijten & van Ham, 2013; Flowerdew and Al‐Hamad, 2004; Mikolai et al., 2019). Having resident children (Cooke et al., 2016 for the United States) or young or school-aged children (Mulder & Malmberg, 2011 for Sweden) is found to lower the probability of long-distance moves following separation. Further, a limited body of literature has addressed spatial mobility in the years after the initial event of separation, and some of these studies extend to include repartnering, but not children’s residential situation (Feijten & van Ham, 2007, 2013). Feijten and Van Ham (2007, 2013) show that separated parents move relatively short distances in comparison with their married and cohabiting counterparts. In addition, some studies have shown that separated parents tend to live close to each other (Thomas et al., 2017a) and, correspondingly, that a large proportion of parents live close to non-resident children (Stjernström & Strömgren, 2012). The distances between members of former families tend to increase when one or both parents repartner (Stjernström & Strömgren, 2012; Thomas et al., 2017a, 2017b).

Although these different literature strands have addressed important dimensions of post-separation mobility, with some studies suggesting a negative effect of family complexity on mobility, no previous study has dug deeper to explicitly explore the relationship between family complexity and internal migration. In this study, we compare separated parents with parents in two-parent families in terms of their likelihood of migrating, using detailed records of parents’ partnership events and statuses. We also investigate the role of the children’s residential situation after separation in the likelihood of migrating. In so doing, we add to the literature on parents’ migration after separation in three ways. First, we distinguish resident children from non-resident children living nearby and those living further away. Second, we examine migration behaviour in the context of repartnering after separation. Third, we distinguish between parents’ partnership events (i.e. the events of separating and repartnering, which involve a move of one or both partners by definition) and partnership statuses (i.e. being single, being repartnered) (see also Clark, 2013 for the different effects of life course events and states on moving).

Using register-based population data from the Netherlands, we have estimated event-history models of the probability of moving at least 50 km within the country. In a first analytical step, we follow individuals in couples from the birth of their first child through potential separation and repartnering; in a second step, we perform a more detailed analysis of those who separate.

The Netherlands is one of few countries that offers detailed longitudinal information on geographical locations and family relations and events of all registered individuals in the country. We focus on long-distance moves because of the large impact these have on one’s daily life in both work and family spheres—migration usually involves a change of workplace and, in this case of separated parents, might involve a change in children’s residential arrangements. In comparison with other European countries, the levels of internal migration in the Netherlands are slightly higher than average—migration rates are typical of North–West Europe, and markedly higher than in South–East Europe (Bell & Charles-Edwards, 2014). As in other countries, migration propensities are strongly age-dependent, and the parents who form our study population are of an average age at which long-distance moving is considerably less common than among younger adults (Kooiman et al., 2018).

## Background

The family migration literature (see Cooke, 2008 for an overview) moved the focal point of migration research from the individual to the immediate family—traditionally, married couples with or without children. Mincer (1978) conceived a family’s decision to migrate or to stay as the result of an assessment of the monetary and non-monetary costs and benefits of migrating for all members of the family, using family as a synonym for household. Today, the increasing plurality and complexity of family life demand a broader conceptualization of the decision-making unit of migration, and of ‘the family’. Yet, we use Mincer’s conceptualization as our starting point in analysing the migration behaviour of parents in a range of family structures: with or without partners and with resident or non-resident children. We formulate five key theoretical arguments that guide our hypotheses. The first two hypotheses concern parents’ partnership status and are tested with a model that includes both separated and non-separated parents. The third, fourth and fifth hypotheses concern children’s residential situation and are tested with a model that includes only separated parents.

### Parents’ Partnership Status

The first argument on which we base our hypotheses about the effect of parents’ partnership status on migration is that the larger a family unit is, the less likely the family is to migrate (Mincer, 1978). Larger families will typically have established more local ties: human, economic and social capital that cannot, or not easily, be relocated.^4^ Examples are ties to work, school, friends and family living nearby. These ties tend to make staying more beneficial than migrating. Also, the risk of ‘locational conflict’ (Cooke et al., 2016) within a family, i.e. conflicting needs and preferences about where to live, is higher when more people are involved in the decision-making (see also Costa & Kahn, 2000). On average, single-parent families form smaller units than two-parent families, with fewer local ties and less potential for locational conflict. In any case, single parents’ family units are smaller by one partner. Assuming that a partner generally has more influence in location decisions than additional children, with distance to a partner’s workplace playing an important role, this means that most single parents can decide more freely where to live. Therefore, we hypothesise that *single parents are more likely to migrate than parents in two-parent families (H1)*.

The second argument is that ex-partners often continue to play a role in separated parents’ migration decisions, despite no longer being members of the household, because the parents have an interest in living close to each other. That is, geographical proximity is critical for sharing childcare responsibilities (Bakker and Mulder, 2013; Stjernström & Strömgren, 2012; Thomas et al., 2017b). Many separated parents who live in close proximity, and particularly those who are involved in a shared residence arrangement,^5^ will consult each other before moving a significant distance. In these situations of active consultation and negotiation, the ex-partner could be conceived of as part of the migration decision-making unit, even though not part of the household. Given the continued linked lives of separated parents, many repartnered parents need to coordinate locational decisions not only with their new partner but also with their ex-partner. We therefore expect that *repartnered parents are less likely to migrate than parents in two-parent families (H2)*. The implication is that repartnered parents are expected to be less likely to migrate than single parents.

A few studies have shown evidence of the continued linked lives of post-separation families. Cooke et al. (2016) found that the post-separation migration decisions of parents are interdependent and argue that this is likely to be due to child visitation and custody arrangements. Similarly, Thomas et al. (2017b) showed that the distance between separated parents is significantly shorter than between ex-partners without shared children. Indeed, based on interview findings, Gram-Hanssen and Bech-Danielsen (2008) reported that parents find it important to live close to the daily life space of their non-resident children and thus, they tend to remain close to their ex-partner. Not surprisingly then, separated fathers are found to move short distances, implying that they wish or need to stay close to their previous place of residence where their children are likely to still live with their mother (Feijten & van Ham, 2007). Thomas et al. (2017b) further found that the distance between ex-partners varied depending on where the children lived and that it was shortest if the children lived with each parent alternately (i.e. shared residence).

Naturally, the events of separating and repartnering, which are key to producing family complexity (Thomson, 2014), are associated with an increased likelihood of migrating. That is, these events almost always, and by definition in our operationalization, require one or both partners to change residence. Although most moves following separation take place over short distances (Mulder & Malmberg, 2011), the propensity for moving over long distances (i.e. migration) is also elevated at the time of separation (Clark & Davies Withers, 2007; Cooke et al., 2016). With the exit of a partner from the migration decision-making unit, new opportunities arise to fulfil individual locational preferences (Cooke et al., 2016). Long-distance moves at the time of union formation are also common. For Sweden, Brandén and Haandrikman (2018) showed that the average distance of moving at the start of co-residence is 50 km for men and 59 km for women. Repartnering upon or after separation was found to increase mothers’ likelihood of moving to another municipality or province within Belgium (Schnor & Mikolai, 2020).

### Child Residence After Parental Separation

The third theoretical argument, on which we build our hypotheses about the role of children’s residential situation among separated parents, is that migration is particularly costly for children, especially when they are of school age (Mincer, 1978; see Webb et al., 2016 for a study on the links between childhood residential mobility and negative outcomes in later life). Parents tend to prioritise stability in their children’s schooling and social network over personal migration opportunities (Bailey et al., 2004). As a result, families with resident children in general (e.g. Fischer & Malmberg, 2001), or with school-aged children specifically (Michielin & Mulder, 2008), are found to be relatively immobile. Parents’ desire to protect children’s local ties to their school, friends and home could be especially strong at the time of, and after, family dissolution. Gram-Hanssen and Bech-Danielsen (2008) show that parents with resident children are likely to stay in the same region and also the same home after separation, aiming to minimise further disruption of their children’s lives following separation. In addition, many separated parents with resident children will consider the other parent (i.e. their ex-partner) in their locational decisions, for the sake of that parent’s continued involvement in their children’s lives.

The fourth argument is that, similar to ex-partners, non-resident children (in general, children living with the other parent) often continue to play a role in separated parents’ migration decisions, despite no longer being members of the household. As most parents value geographical proximity to their children, a non-resident child who lives nearby can be seen as a local tie and thus a cost of migration for the parent (see Mulder, 2018; Mulder & Malmberg, 2014) or, as Schewel (2019) phrased it, a social retain factor that bolsters a preference to stay. Arguably, parents of non-resident children living nearby face even greater costs of migrating than parents with resident children. For them, migrating equals moving away from their children if the ex-partner stays in the same location with the children. In sharp contrast, parents with non-resident children living further away are unlikely to face any child-related costs of migrating, and some could in fact be motivated to migrate to be closer to their children (see Gillespie & Mulder, 2020 for a study on non-resident family as a motive for migration). Further, for many of these parents, the significant distance to their children will be the outcome of an earlier migration decision. Therefore, longer distances may be an indication that the parent is mobility prone in general, or that children form a less central consideration in the parent’s locational decisions.

Based on the third and fourth argument, we hypothesise that *in comparison to having resident children, having non-resident children living further away is associated with a higher likelihood of migrating (H3a), and having non-resident children living nearby is associated with a lower likelihood of migrating (H3b)*.

It is important to note that a substantial share of the parents with non-resident children living nearby will be involved in a shared residence arrangement (see, for example, Skjørten et al., 2007; Thomas et al., 2017a), as well as some of the parents with resident children. In the situation of shared residence, parents will be very unlikely to migrate. For example, Ferrari et al. (2019) show that recently divorced parents with joint physical custody move shorter distances away from the prior joint home than non-custodial or sole custody parents. Shared residence parents have typically made an explicit agreement in a parenting plan about a maximum moving distance (Bakker and Mulder, 2013). If these parents migrate, they will probably need to change their residential arrangement due to the significant increase in geographical distance. Unfortunately, we cannot identify shared residence in our data (see footnote 5).

Another source of family complexity is the formation of stepparent-stepchild, stepsibling and half-sibling relationships through repartnering. Based on our first argument on family size, we hypothesise that, in addition to any effects of one’s own children, *having new joint children*^6^* or stepchildren is associated with a lower likelihood of migrating (H4)*. These children imply a larger family unit with increased potential for locational conflict and additional costs of migrating for the family due to local ties.

### Gender Differences

The fifth argument is that women might be more inclined than men to rank family over other life spheres, such as work, as a result of gendered expectations and divisions of paid work and childcare (see, for example, Maume, 2006). As a consequence, women may be less receptive to opportunities offered by migrating, e.g. career advancement, if this compromises the family sphere. Conversely, they may be more motivated to migrate if this benefits the family sphere. Previous research has shown evidence of such gendered differences. For instance, Thomas et al. (2017a) found that the distance between parental ex-partners is smaller when the children live with the father than when they live with the mother, suggesting that mothers with non-resident children find proximity more important than fathers.

In the Netherlands, traditional, gendered ideas about the division of labour and childcare are still reflected in social norms about demographic behaviour (Liefbroer & Billari, 2010). Also, the state’s family policies can be seen as supportive of a traditional gendered division of labour, providing mostly general family support (e.g. cash child allowances) rather than dual-earner support (Lundberg et al., 2008). Correspondingly, Dutch women spend considerably more time on childcare than men and, also in comparison with other OECD countries, this balance is heavily skewed (The Fatherhood Institute, 2010). By the same token, women in the Netherlands remain far more likely than men to be the children’s primary caregiver after separation. However, most fathers stay in frequent contact with their non-resident children (Spruijt & Kormos, 2014) and remain involved in their lives (Westphal et al., 2014). In addition, there has been an increase in shared residence arrangements over recent decades in which children spend equal or near-equal time at both parents’ homes (Poortman & van Gaalen, 2017). Shared residence has been promoted by Dutch legislation^7^ since 2009. In 2013, 22.2% of parents opted for shared residence at the time of divorce, compared with 70.4% opting for children living with the mother and 7.4% opting for children living with the father (Poortman & van Gaalen, 2017).

Given gendered expectations and divisions of childcare, we hypothesise that *the positive effect of having non-resident children living further away on parents’ likelihood of migrating (H5a), and the negative effect of having non-resident children living nearby (H5b), rather than resident children only, is stronger for women than for men*.

The literature on post-separation mobility has also revealed other gender differences. All else being equal, women seem to be more likely than men to move out of the joint home (Mulder & Wagner, 2010, 2012) and migrate (Cooke et al., 2016) after separation. Among couples with joint children, women are less likely to move than men because the children often stay to live with the mother rather than with the father (Ferrari et al., 2019; Gram-Hanssen & Bech-Danielsen, 2008; Mulder & Malmberg, 2011; Mulder & Wagner, 2010). Separated, single men with children are found to move over considerably shorter distances than separated, single women with children, because these men are tied to their previous place of residence where the children typically still live with their mother (Feijten & van Ham, 2007). In the event of union formation, women are more likely than men to move or migrate, although the presence of children lowers this likelihood (Brandén & Haandrikman, 2018).

## Data, Measures and Methods

### Dataset and Study Population

We used annual data from the System of social statistical datasets (SSD) of Statistics Netherlands (Bakker et al., 2014). Combining several administrative registers, the SSD contains longitudinal information on the entire population of the Netherlands, including information on locations and distances and also record linkages between family members.

Our study population comprises all individuals who, between the ages of 18 and 50, had their first child in 2002, 2003 or 2004 within a cohabiting, married or registered partnership. These individuals are referred to as anchors from here onwards. We followed these anchors from the first year of being in a two-parent family to the end of the observation period, regardless of whether they remained in the two-parent family, became single or repartnered. Observation started in the year following the birth of this first child and continued until 2015, that is, until the child’s age was between 10 and 12 (primary school age or younger). Observations were right-censored at the end of the final year before the anchor died, emigrated, entered an institutional household or dissolved the first repartnered union after the start of the observation.

By selecting parents of first children born in 2002, 2003 or 2004, we limited variation between parents in terms of their children’s ages (from birth till primary school age) and the time period covered. In addition, data on income were only available from 2002 onwards, and data on education were most complete for these more recent years. We only selected those parents for whom their first child was also their partner’s first-born. In this way, observation starts with all anchors in the same stage of the life course, namely in a ‘simple’ two-parent family without possible stepparent-stepchild relations, and we avoid problems of left-censoring. Moreover, we eliminated the possibility of anchors having children with more than one ex-partner by setting this initial condition and by censoring the observations after dissolution of the first repartnered union. Including third and subsequent unions would add an extra layer of complexity to parents’ migration decisions that we could not adequately capture in our analyses. Both parents are included in the analyses as anchors, provided that they both met the age requirement.

After excluding some further observations, mostly due to missing location data (see Appendix 1 for more information), our final dataset contained 5,043,641 person-years of 220,809 men and 221,603 women. During the window of observation, 22,547 internal migration events occurred to men and women combined.

### Dependent Variable

The dependent variable is whether or not an internal migration event occurred during a calendar year (*t*), with migration defined as a change of address within the country of at least 50 km. A cut-off distance of 50 km is commonly used, across a range of countries (e.g. Boyle et al., 2001 for Great Britain and the USA; Clark & Lisowski, 2019 for Australia; Mulder & Malmberg, 2014 for Sweden). Moves over such distances are likely to involve a change of workplace and will have a serious impact on resident children’s daily lives and on the frequency of face-to-face interactions between parents and non-resident children. In Sect. 4.5, we report the results of sensitivity checks on the distance threshold. Given that we used annual data, comparing addresses on 1 January of year *t* and *t* + 1, we could not account for multiple migration events within one year.

### Key Predicting Variables

The first key predicting variable is the anchor’s partnership status. This variable consists of the following seven categories, four of which represent statuses (situation is the same on 1 January of year t and *t* + 1) and three of which represent events (situation changes between 1 January of year *t* and *t* + 1):

1. *In two-parent family* Status, anchor lives in the initial two-parent family, with the other parent of one’s first-born child.
2. *Separating* Event, anchor’s situation changes from being in the initial two-parent family to being single.
3. *Separating & repartnering* Event, anchor’s situation changes from being in the initial two-parent family to being repartnered.
4. *Separated last year* Status, anchor separated in the previous calendar year and is single.
5. *Separated & single* Status, anchor separated more than one year ago and is single.
6. *Repartnering* Event, anchor’s situation changes from being single to being repartnered.
7. *Repartnered* Status, anchor lives with a first new partner since dissolution of the initial two-parent family.

We distinguished parents who separated in the last year (category 4) from single parents who separated longer ago (category 5) to account for the elevated risk of moving following the initial separation event (see, for example, Feijten & van Ham, 2013; Mikolai & Kulu, 2018). Separation is defined as living at separate addresses for at least 365 days (see Appendix 1 for more information). See Table 1 for the distribution of this and other independent variables.

**Table 1:** Descriptive statistics for the population of separated and non-separated parents, column percentages of person-years and counts of migration events

	Men		Women	
Number of person-years	2,504,254		2,539,387	
Number of individuals	220,809		221,603	
Number of migration events	11,101		11,446	
	% of PY	Events	% of PY	Events
*Partnership status*				
In two-parent family	91.65	8566	90.72	8610
Separating	1.40	719	1.45	1035
Separating & repartnering	.18	287	.14	270
Separated last year	1.08	275	1.21	224
Separated & single	3.02	449	4.04	436
Repartnering	.56	651	.49	726
Repartnered	2.11	154	1.96	145
*Child residence situation *^a^				
Resident child(ren) only	8.16	65	88.95	1153
Near non-resident child(ren)	39.40	369	2.07	27
Far non-resident child(ren)	33.14	737	1.59	79
Resident & near non-resident children	11.23	28	5.90	30
Resident & far non-resident children	8.06	55	1.49	18
*Resident stepchildren or joint new children *^a b^				
Yes	4.76		.87	
No	95.24		99.13	
*Ties to own and partner’s parents*				
None nearby or resident	35.93	8035	36.04	8282
At least one parent resident	2.34	2529	2.08	2788
Parent nearby	61.73	537	61.88	376
*Any children of primary school age*				
All children age < 4	34.46	6567	34.19	6855
At least one child of primary school age (4–12)	65.54	4534	65.81	4591
*Age*				
19–29	7.56	1550	16.12	3023
30–34	22.87	3576	29.88	4190
35–39	33.00	3569	31.91	2989
40–44	24.94	1767	17.60	1027
45–62	11.64	639	4.49	217
*Period*				
2003–2008	43.02	7756	42.72	8036
2009–2015	56.98	3345	57.28	3410
*Living in municipality of birth*				
Yes	78.08	1020	79.18	883
No	21.92	10,081	20.82	10,563
*International migrant status *^c^				
Native Dutch	82.70	8812	81.48	8822
First-generation immigrant	10.58	1404	11.43	1649
Second-generation immigrant	6.71	885	7.08	975
*Housing tenure*				
Owner-occupied	80.22	6926	78.33	6811
Private rental	2.10	567	2.39	551
Social rental	9.17	611	10.83	834
Unspecified rental	7.09	2766	7.07	3016
Unknown housing tenure	1.42	231	1.37	234
*Completed education*				
Intermediate vocational training or lower	74.04	6444	72.06	6818
Undergraduate degree	16.05	2102	17.88	2365
Graduate degree	9.91	2555	10.06	2264
*Employment status*				
Employed	76.96	8369	70.35	6516
Self-employed	16.28	1393	8.26	878
Student	.15	72	.54	160
Not employed	6.61	1267	20.85	3892
*Equivalised household income, deciles of full population*				
Income unknown	.17	115	.19	131
0–10%	4.72	796	5.68	1172
10–20%	5.78	659	6.72	915
20–30%	7.89	714	8.34	875
30–40%	10.83	922	11.08	983
40–50%	13.25	1015	13.16	1039
50–60%	13.68	1128	13.36	1102
60–70%	12.69	1273	13.24	1167
70–80%	11.23	1343	10.71	1258
80–90%	10.13	1437	9.54	1314
90–100%	9.63	1699	8.98	1490
*Urbanity of municipality*				
Not urban	9.26	667	9.23	696
Hardly urban	22.61	1646	22.52	1698
Moderately urban	18.32	1711	18.31	1720
Strongly urban	31.04	3855	31.14	4008
Very strongly urban	18.76	3222	18.81	3324
*Jobs* < *50 km of municipality*				
< 1/2 million	19.45	2086	19.43	2174
1/2–1 million	24.17	2294	24.17	2326
1–2 million	34.37	3736	34.31	3874
2 + million	22.01	2985	22.09	3072
*Avg housing costs per m2 in municipality*				
< 2000	26.25	2744	26.21	2849
2000–2250	33.66	3524	33.69	3662
2250–2500	13.35	1542	13.29	1549
2500 +	26.74	3291	26.80	3386
*Ex-partner in new union *^a^				
Yes	29.39	324	33.64	333
No	70.61	930	66.54	974
*Mobility at separation *^a c^				
Stayed	50.21	438	39.08	485
Moved out	49.79	816	60.92	822
*Mean duration since separation (years)* ^a^	3.46		3.68	

*SSD* Statistics Netherlands (own calculations)

^a^ Only for separated parents, who are single, repartnering or repartnered (Model 2 population)

^b^ Following the privacy regulations of Statistics Netherlands, these event counts are not reported because of low frequencies (*n* < 10)

^c^ Time-constant variable; all other variables are time-varying

The second key predictor is the residential situation of the first child and any other children who were born in the same union as this first child, as observed on 1 January of year *t*. This variable is used in a model including separated parents only (i.e. parents who are single, repartnering or ‘first-time repartnered’). The variable has five categories: resident child(ren); near non-resident child(ren); far non-resident child(ren); resident & near non-resident children; resident & far non-resident children (see Appendix 1 for more detailed information on this classification). Anchors’ children are classified as living nearby when living within 5 km; when a child lives more than 5 km away, the child is classified as far non-resident. The choice for this distance threshold was based on earlier research that showed that parents and children are more likely to exchange instrumental support when living within 5 km of each other rather than further away (Knijn & Liefbroer, 2006). In our dataset, 60.8% of all parents with a non-resident child lived within 5 km of the child living closest. See Sect. 4.5 for sensitivity checks on this distance threshold.

Unfortunately, children residing with both parents on a fairly equal basis (i.e. shared residence) cannot be identified from the Dutch register data because parents can only register a child at a single address. In such situations, the choice of with which parent to register a child is sometimes fiscally motivated (for example, one child is registered with the father and one with the mother to improve both parents’ likelihood of qualifying for certain single parent tax benefits) and may even be random (van der Wiel & Kooiman, 2019). Yet, from an analysis of survey data matched to the register data, Van der Wiel and Kooiman (2019) concluded that, for the vast majority of children, the registered address matches their main place of residence as reported by one of their parents. Most parents with shared residence for their children live in close proximity to each other (see Bakker and Mulder, 2013; Skjørten et al., 2007), so that the children can easily access their school and social network from both places of residence. The implication for this study is that, although we cannot tell from the data, it is likely that a significant minority of parents with resident children and/or non-resident children living nearby will in fact be in a shared residence arrangement. In contrast, only a very small minority of parents with non-resident children living further away will be in a shared residence arrangement.

The third key predictor, also exclusively for the model with separated parents, is a dummy variable indicating the presence of a resident stepchild or a new joint child on 1 January of year *t*. Stepchildren and new joint children are combined in one variable because there were too few migration events among those with a new joint child.

Overall, 18% of the anchors in our study population separated from the other parent of their first child and 42% of these separated parents repartnered within the window of observation. Consequently, most person-years are spent in two-parent families (92% for men; 91% for women). The vast majority of separated parents with resident children only (i.e. resident but no non-resident children) are women (93%), and the majority of parents with non-resident children only, living nearby or further away, are men (94% and 95%, respectively).

### Control Variables

We took account of proximity to parents (indicated by whether the anchor’s parents and/or the anchor’s partner’s parents live within 5 km or in the same household), housing tenure and living in one’s municipality of birth (see also Mulder & Malmberg, 2014). To account for children’s local ties to school, we distinguished anchors who have a child of primary school-age (4–12 years) from those with younger children only. We distinguished natives from first- and second-generation immigrants. We further accounted for the previous year’s (*t *− 1) equivalised household income in deciles of the full population, educational attainment and employment status as indicators of resources (see, for example, Faggian et al., 2015; Lundholm, 2007; Mulder & Malmberg, 2014). The anchor’s age is taken into account since migration is highly age-specific (Bernard et al., 2014). We distinguished two periods, 2003–2008 and 2009–2015, because the financial crisis that lasted from roughly 2008–2016 in the Netherlands is likely to have had an impact on internal migration. Further, the 2009 change in Dutch legislation (see footnote 4) might have led more parents to actively share parental responsibilities after separation. As contextual factors, we considered the urbanity of the anchor’s municipality (based on the number of addresses per square km) (Mulder & Malmberg, 2014), housing prices (average housing costs per square meter in the anchor’s municipality) and employment opportunities (number of jobs^8^ within a 50 km radius of the centroid of the anchor’s municipality) (Thomas et al., 2017b).

An additional set of control variables is included in the model for separated parents only. We accounted for whether the ex-partner is in a new union (see Thomas et al., 2017a) and included duration since separation (in years) and an indicator of whether the anchor moved out of the joint home at the time of separation (e.g. Mikolai & Kulu, 2018). Unless specified otherwise, all control variables described above were measured and updated on 1 January of the year of observation (t), so prior to any migration event during year t.

### Analytical Strategy

We conducted discrete-time event-history analysis to study the likelihood of internal migration, employing logistic regression of person-years (Yamaguchi, 1991). We estimated two main models, separately for men and women. The first model covers the full study population (*n* = 220,809 men, *n* = 221,603 women). This model is used to explore how separated parents’ likelihood of migrating compares to that of non-separated parents, as a way of comparing ‘complex’ to ‘simple’ (two-parent) families. The second model includes separated parents only (*n* = 31,360 men, *n* = 33,404 women) and is used to study the effects of several separation-specific variables, including child residence and the presence of stepchildren and/or new joint children, on migration. See Appendix 2, Table 4 for the descriptive statistics of this model. Robust standard errors were calculated to adjust for the possible clustering of migration events within individual parents.^9^

Additionally, to statistically test for gendered differences in the effect of child residence and partnership status on parents’ likelihood of migrating, we performed analyses where we pooled the data for men and women and included interaction terms between gender on the one hand and child residence and partnership status on the other. In these interaction models, standard errors were adjusted for the clustering of men and women in the two-parent family in which the first child was born.

## Results

### Parents’ Partnership Status

Table 2 presents estimated odds ratios of migrating relative to not migrating for parents with different partnership statuses and events, all compared to parents who remain in their two-parent family, i.e. living with their first child(ren) and the other parent. Not surprisingly, parents’ likelihood of migrating is significantly higher in the event of separating, by a factor (odds ratio—OR) of 5.66 for men and 7.98 for women (*p* = .00 for both; from here on, *p*-values are only reported if >  = .05). Single parents who separated in the previous year and those who have been separated and single for longer are 1.45–2.06 times more likely to migrate than parents in two-parent families. This finding is in line with the argument that larger family units are less likely to migrate and supports Hypothesis 1.

**Table 2:** Logistic regression results for migration by separated and non-separated parents, presented as odds ratios relative to not migrating—key variables only

	Men				Women				Gender diff
	OR	Z	95% CI		OR	Z	95% CI		*p*
*Partnership status (ref. in two-parent family)*									
Separating	5.66***	42.17	5.22	6.13	7.98***	58.88	7.45	8.55	.000
Separating & repartnering	16.80***	39.82	14.62	19.30	22.67***	42.10	19.60	26.22	.002
Separated last year	2.06***	11.39	1.82	2.33	1.45***	5.21	1.26	1.67	.000
Separated & single	1.82***	10.49	1.62	2.03	1.52***	7.28	1.36	1.70	.001
Repartnering	11.05***	51.55	10.09	12.11	16.66***	57.84	15.14	18.33	.000
Repartnered	1.15	1.58	.97	1.37	1.32***	3.09	1.11	1.58	.274

*SSD* Statistics Netherlands (own calculations)

Partnership status is time-varying. See Appendix 2, Table 5 for the control variables of this model. Test for gender difference based on model with interaction term

*OR* odds ratio; *Z* Z statistic for testing OR = 1

^†^*p* < .10; *p < .05; **p < .01; ***p < .001

Parents’ likelihood of migrating is even more highly elevated in the event of repartnering (men: OR = 11.05; women: OR = 16.66) than in the event of separating, suggesting that long-distance moves are more common when repartnering than when separating. Parents who experienced separating from the other parent of their children and repartnering during the same calendar year seem to be a specific group that is particularly likely to migrate during this year (men: OR = 16.80; women: OR = 22.67). This finding is consistent with that of Schnor and Mikolai (2020), who showed that repartnering at separation lowers mothers’ likelihood of staying in the prior joint home and increases their likelihood of moving to another municipality or province. It seems likely that many of these parents initiated the separation because of their new romantic involvement and consequently moved out. Indeed, previous research has shown that the ex-partner who initiates the separation is considerably more likely to move out of the joint home than the other partner (Mulder & Wagner, 2010).

Once repartnered, fathers’ likelihood of migrating appears to be very similar to that of fathers in two-parent families (OR = 1.15, *p* = .11). Repartnered mothers are somewhat more likely to migrate than their counterparts in two-parent families (OR = 1.32). Thus, contrary to our expectations (H2), it does not appear that repartnered parents are less likely to migrate than parents in two-parent families, despite the family complexity associated with repartnering. It is possible that effects of selection into repartnering explain this unexpected finding: Stability in their children’s lives, also in terms of location, may be a less central concern for parents who repartner than for other parents.

### Child Residence After Parental Separation

In the next step, we focus on the parents who are separated and look at how their likelihood of migrating differs with their child residence situations (see Table 3). In line with Hypothesis 3a, we found that having children who live further away than 5 km is associated with a much higher likelihood of migrating than having resident children (men: OR = 1.78; women: OR = 2.64). Parents with non-resident children living further away are unlikely to face any child-related costs linked to migrating. Moreover, these parents may be mobility prone, considering that the distance to their children will for many be the outcome of an earlier migration decision. Additionally, some parents will in fact migrate to live closer to their children. Indeed, of those who migrated among these parents, 43% of fathers and 53% of mothers lived closer to their child(ren) after migrating (distance decreased by at least 1 km). These proportions are even higher among parents who are single (i.e. not having repartnered): 53% of fathers and 64% of mothers. This finding suggests that living closer to children is a common consideration in migration decisions of separated parents, slightly more for mothers than for fathers, and in particular for those who are single. Furthermore, our results suggest that, for men, having both resident children and non-resident children living further away may also be associated with a higher likelihood of migrating than having resident children only (OR = 1.47; *p* = .06); but that this is not the case for women (OR = .90; *p* = .68).

**Table 3:** Logistic regression results for migration after separation, presented as odds ratios relative to not migrating—key variables only

	Men				Women				Gender diff
	OR	Z	95% CI		OR	Z	95% CI		*p*
*Partnership status (ref. separated & single)*									
Repartnering	6.66***	26.02	5.78	7.69	12.25***	33.98	10.60	14.15	.000
Repartnered	.60***	− 4.53	.48	.75	.81†	− 1.83	.64	1.01	.019
*Child residence situation (ref. resident child(ren))*									
Near non-resident child(ren)	.78	− 1.63	.59	1.05	.74	− 1.49	.49	1.10	.532
Far non-resident child(ren)	1.78***	3.97	1.34	2.37	2.64***	6.99	2.01	3.46	.168
Resident & near non-resident children	.41***	− 3.80	.26	.65	.38***	− 4.99	.26	.56	.613
Resident & far non-resident children	1.47†	1.91	.99	2.20	.90	− .41	.54	1.49	.059
*Step- or joint new children*	.40*	− 2.02	.04	.16	1.84	1.51	.74	1.51	

*SSD* Statistics Netherlands (own calculations)

These three variables are time-varying. See Appendix 2, Table 6 for the control variables of this model. Test for gender difference in partnership status and child residence based on model with interaction term

*OR* odds ratio; *Z* Z statistic for testing OR = 1

^†^*p* < .10; *p < .05; **p < .01; ***p < .001

Hypothesis 3b was not supported in that no significant difference was found between having non-resident children living nearby (within 5 km) and having resident children, although separated parents’ likelihood of migrating was estimated to be lower for those with non-resident children nearby (men: OR = .78, *p* = .10; women: OR = .74, *p* = .14). Nevertheless, a noteworthy finding is that having both resident children and non-resident children living nearby is associated with a significantly lower likelihood of migrating than having resident children only (men: OR = .41; women: OR = .38). In some of these cases, one or more children will indeed live with the mother and the other child(ren) with the father. Other parents with resident and near non-resident children will be in shared residence arrangements, having registered one child with each parent. In both situations, many of these parents are probably unwilling to disrupt the lives of their children by migrating away from the other parent and possibly the child’s siblings.

We had expected that having new joint children with, or resident stepchildren from, the new partner would be associated with a lower likelihood of migrating (H4). While this is true for men (OR = .40), we found no evidence supporting this idea for women (OR = 1.84, *p* = .131). Lastly, Table 3 confirms that repartnered parents are less likely to migrate than single parents (men: OR = .50; women: OR = .81, *p* = .07), signalling the effect of the new partner as a source of added locational conflict and additional local ties (see Sect. 4.1).

### Gender Differences

The far-right column in Table 2 shows the results for the interaction between gender and partnership status. Overall, it seems that women are considerably more likely than men to migrate in event years, i.e. in the year of separating and/or repartnering, and a little less likely to migrate once separated. Previous studies have also reported on women’s relatively high likelihood of migrating upon union formation (Brandén & Haandrikman, 2018 for Sweden) and union dissolution (Cooke et al., 2016 for the USA).

The results for the interaction between gender and child residence are shown in the far-right column of Table 3. Although the estimated positive effect on migration of having non-resident children living further away is stronger for women than for men (men: OR = 1.78; women: OR = 2.64), the gendered difference in this effect is not statistically significant (*p* = .168), leaving Hypothesis 5a unsupported. Further, we found no evidence for a gendered difference in the effect of having non-resident children nearby on parents’ likelihood of migrating (H5b, *p* = .532).

### Control Variables

The effects of the other local ties, resources and contextual factors considered are shown in Tables 5 (results for the full study population) and 6 (separated parents only) in Appendix 2. Our discussion on these effects is based on Table 5 unless stated otherwise. Both men and women are much less likely to migrate if a parent or partner’s parent lives nearby, indicating the costs of migrating away from family. In the full study population, co-residing with a parent is also associated with a lower likelihood of migrating, but less so than having a parent nearby. Among the subpopulation of separated parents only (Table 6), co-residing with a parent increases a man’s likelihood of migrating. It seems likely that most of these separated men moved back in with a parent temporarily for shelter or support. Having a child of primary school age (4–12) lowers the likelihood of migrating by about 20–25% for the full study population, most of whom are in two-parent families. Among the subpopulation of separated parents only (Table 6), having a child of primary school age has a very small effect on migration, but it is estimated to be positive.

Older men and women are less likely to migrate than younger ones. Further, the likelihood of migrating in the period 2009–2015 (during an economic recession) was about half that of the earlier 2003–2008 period. Men and women who live in the municipality of their birth are about half as likely to migrate as those who do not. First-generation immigrants are less likely to migrate than natives. Compared to persons living in owner-occupied housing, those in private rental accommodation are much more likely to migrate, while those in social housing are somewhat less likely to migrate. In comparison with those whose education did not extend beyond intermediate vocational training at best, men and women with an undergraduate degree and especially those with a graduate degree are more likely to migrate. Students and non-employed people are more likely to migrate than those who are employed. Self-employed men are less likely to migrate than their employed counterparts, whereas self-employed women are more likely to migrate. The coefficients for income suggest a U-shaped relationship with the likelihood of migrating. Further, the more urban a municipality, the greater the likelihood of migrating, but the availability of more jobs in the region is associated with a somewhat lower likelihood of migrating. Higher average local housing costs in the municipality are associated with a lower likelihood of migrating.

Three additional control variables specific to the population of separated parents are included in the model reported in Table 6. For every additional year since separation, men’s likelihood of migrating is estimated to drop by 5%. Men who moved out of the joint home at the time of separation are more likely to migrate in the years following than men who stayed in the home. However, duration since separation and whether one moved from the joint home have no significant migration effect for women. Whether the ex-partner is in a new union is not significantly related to a separated parent’s migration.

### Additional Analyses

We performed sensitivity analyses for the distance thresholds used to define migration and to distinguish near from far non-resident children. These analyses showed very similar results for migration defined as moves over at least 30 or 40 km compared with 50 km. Using 2 km instead of 5 km as the threshold for children living nearby also led to similar results, but the differences between parents with non-resident children living nearby versus further away were more evident using the 5 km threshold. Furthermore, the results were robust to alternative restrictions of age at first childbirth (age 20–40 or 25–35). Our selection of anchors aged 18–50 excludes outliers but is otherwise fairly inclusive.

An additional interaction model did not show that the effect of children’s residence situation on parents’ likelihood of migrating depends on the parent’s partnership status, with one exception: the association between mothers’ likelihood of migrating and having non-resident children living further away, as opposed to having resident children, is less positive for repartnering mothers than for single mothers (OR = .35).

## Conclusions and Discussion

In recent years, it has been suggested that increasing family complexity may be a factor in declines in internal migration (Cooke et al., 2016; Thomas et al., 2017a). The relationship between family complexity and individual migration behaviour has, however, never been studied explicitly. In this study, we compared separated parents with parents in two-parent families as to their likelihood of migrating (moving at least 50 km), using detailed records of parents' partnership status and children's residential situations. We used longitudinal population register-based data for couples in unions who became first-time parents in 2002, 2003 and 2004 in the Netherlands. We have made three contributions to the literature on parents’ migration following separation. First, we distinguished between resident children and non-resident children living nearby and further away. Second, we examined migration behaviour in the context of repartnering after separation. Third, we distinguished between partnership events and statuses.

In contrast to the suggestion that increasing family complexity may lead to falling rates of migration, based on our findings, one would expect family complexities stemming from parental separation to be associated with increased levels of migration. Naturally, the events of separating and repartnering in themselves lead to increased risks of migrating. At the same time, single mothers and fathers, as well as repartnered mothers, are also more likely to migrate than their counterparts in two-parent families. Repartnered fathers’ likelihood of migrating appears similar to that of fathers in two-parent families.

Our analyses further show that parents’ likelihood of migrating after separation depends heavily on where their children live. That is, having non-resident children who live further away is associated with a much higher likelihood of migrating than having resident children or non-resident children living nearby. Having both resident children and non-resident children who live nearby is associated with a considerably reduced likelihood of migrating than having resident children only. These findings highlight the need to distinguish non-resident children living nearby from those living further away. Ties to non-resident children can pose a constraint on migration (see Thomas et al., 2017a), but only when the children live nearby. Conversely, children who live further away seem to draw many parents to move closer to them. Many parents with both resident and non-resident children nearby will be unwilling to disrupt the lives of their children and any residential arrangement for their children that they have with their ex-partner—with shared residence likely to be common in this group. As such, our finding supports Cooke et al.’s (2016) notion that shared (physical) custody (i.e. shared residence) may be associated with reduced levels of migration. Hence, if shared residence after separation becomes more common in the future, which is not unlikely, this might lead to lower migration levels.

It should be borne in mind that our data do not contain information about the residential arrangements of post-separation families, nor about the frequency of contact between parents and non-resident children. Rather, we based our analyses on children’s registered address and the distance between parents and non-resident children. Previous research has shown that the registered address corresponds by and large to the main place of residence of children of separated parents (van der Wiel & Kooiman, 2019). Even so, there will be some children whose registered address incorrectly identifies which parent they live with, as well as a significant number of children who live half the time with each parent. Also, the reliability of registered addresses depends on inhabitants accurately registering their changes of address. This might not always be the case, particularly in turbulent times following separation. Another limitation of the data is that it is impossible to include international migration. Furthermore, even though population-based register data offer large numbers and thus better opportunities for studying complex family situations than most surveys do, we occasionally reached the limits of our data in terms of the numbers of observations of less common situations. Finally, our choice to start the observation at the time of the birth of the first child (which we deemed important to avoid initial-condition problems and thus left censoring) and censor those who separated for a second time (which we had to do because the numbers would become too small and the number of possible situations would become too large) implied that we did not observe the most complex families: those with children from more than one previous partner.

It would be valuable to conduct similar research in other contexts, where the care for children after separation is arranged differently and divided between mothers and fathers more evenly than in the Netherlands, with its gender normative climate. It would also be useful to replicate this study using information on actual post-separation residential arrangements, rather than official address information, with survey data including sufficiently large numbers of observations. Such survey data might also shed some light on the relatively small groups of mothers living at some distance from their non-resident children and of fathers with resident children.

Overall, through our comprehensive analyses of parents’ migration after separation, we have been able to add nuanced evidence to the discussion on the effects of increasing family complexity on internal migration and show that ties to children play an important role in separated parents’ migration decisions. Altogether, our findings suggest that many parents prioritise proximity to non-resident children, their children’s sibling relations and/or the relationship between their ex-partner and children over other opportunities that migration might offer them personally.

## Appendix 1 Data preparations details

Person-years were excluded from the analyses if none of the children born in the two-parent family was still alive (5563 person-years). Furthermore, we excluded a small proportion of person-years for which information was incomplete (less than .5% of the total of just over 5 million person-years). For example, we excluded person-years in which one or more children lived at an unknown distance (*n* = 1104). In addition, 15,257 person-years were excluded in which one or more of an anchor’s children did not have a registered address in the Netherlands, nor a registered date of death. Most of these children will live abroad, but some may have died after emigration with their death not recorded. We further excluded 4233 person-years in which the distance to one or more of the anchors’ or anchors’ partner’s parents was unknown because the parents’ place of residence was missing, and 448 person-years in which the anchor’s own address coordinates were missing. Some anchors with strong inconsistencies between records were removed (*n* = 95); these inconsistencies mostly concerned data on partnerships. Finally, we removed eight anchors without registered gender.

Couples who were registered as living at different addresses for under 365 days were not counted as having separated. This regularly occurred in a year of moving, suggesting that the partners registered their new address on different dates, or perhaps that one partner moved ahead of the other. Some parents got back together after having lived apart for at least 365 days. In this case, they were censored at the start of the year of separation, as it was unclear whether these couples had really ended their relationship or were merely temporarily living apart.

To ensure that the categories of the key predicting variable of child residence were mutually exclusive, a few very small categories were combined with other categories. A small number of parents had non-resident children both nearby and further away, and these were included in the category ‘near non-resident child(ren)’. Another small number of parents had resident children, near non-resident children and far non-resident children, and were included in the ‘resident and near non-resident child(ren)’ category.

Only children who were born in the first family were included when classifying child residence. Joint children with a new partner would almost exclusively be resident children and would arguably have a different effect on a couple’s migration decisions than an anchor’s own children, who could be resident or non-resident. In the rare situation of children who were born after the dissolution of the first family but outside an observed co-residential union, these children were included in the classification as they are the parent’s own child and would similarly affect the process of repartnering as children from the first family. However, to maintain consistency with how we treated children born with a new partner, children born outside a union whose other parent was the person with whom the anchor repartnered were not included in the classification of child residence.

## Appendix 2 Additional tables

See Tables 4, 5, 6.

**Table 4:** Descriptive statistics for the population of separated parents, column percentages of person-years

	Men	Women
Number of person-years	142,405	164,720
Number of individuals	31,360	33,404
Number of migration events	1254	1307
*Partnership status*		
Separated & single	53.10	62.26
Repartnering	9.83	7.60
Repartnered	37.07	30.15
*Child residence situation*		
Resident child(ren) only	8.16	88.95
Near non-resident child(ren)	39.40	2.07
Far non-resident child(ren)	33.14	1.59
Resident & near non-resident children	11.23	5.90
Resident & far non-resident children	8.06	1.49
*Resident stepchildren or joint new children*		
Yes	4.76	.87
No	95.24	99.13
*Ties to own and partner’s parents*		
None nearby or resident	46.14	43.84
At least one parent resident	47.25	53.33
Parent nearby	6.61	2.83
*Any children of primary school age*		
All children age < 4	7.45	6.96
At least one child of primary school age (4–12)	92.55	93.04
*Age*		
19–29	5.24	11.69
30–34	16.22	24.84
35–39	31.25	33.64
40–44	30.93	22.91
45–62	16.37	6.93
*Period*		
2003–2008	14.04	13.47
2009–2015	85.96	86.53
*Living in municipality of birth*		
Yes	25.31	24.76
No	74.69	75.24
*International migrant status * ^c^		
Native Dutch	80.83	79.02
First-generation immigrant	10.23	10.81
Second-generation immigrant	8.94	10.17
*Housing tenure*		
Owner-occupied	64.23	42.92
Private rental	7.98	9.94
Social rental	21.04	40.44
Unspecified rental	4.70	5.33
Unknown housing tenure	2.05	1.37
*Completed education*		
Intermediate vocational training or lower	81.97	78.83
Undergraduate degree	12.22	14.69
Graduate degree	5.81	6.48
*Employment status*		
Employed	68.90	66.12
Self-employed	15.78	6.43
Student	.14	.81
Not employed	15.17	26.63
*Equivalised household income, deciles of full population*		
Income unknown	.50	.47
0–10%	6.08	16.06
10–20%	6.34	20.03
20–30%	6.12	13.35
30–40%	8.04	12.98
40–50%	10.19	10.15
50–60%	12.01	8.26
60–70%	12.55	6.40
70–80%	12.56	5.07
80–90%	12.76	4.05
90–100%	12.84	3.19
*Urbanity of municipality*		
Not urban	7.67	7.74
Hardly urban	18.94	18.72
Moderately urban	16.41	16.90
Strongly urban	34.55	34.99
Very strongly urban	22.44	21.65
*Jobs* < *50 km of municipality*		
< 1/2 million	21.25	21.26
1/2–1 million	23.02	23.09
1–2 million	34.74	34.50
2 + million	21.00	21.15
*Avg housing costs per m2 in municipality (ref.* < *2000)*		
< 2000	27.35	27.31
2000–2250	34.43	34.66
2250–2500	13.11	12.61
2500 +	25.11	25.42
*Ex-partner in new union*		
Yes	29.39	33.64
No	70.61	66.54
*Mobility at separation * ^c^		
Stayed	50.21	39.08
Moved out	49.79	60.92
*Mean duration since separation (years)*	3.46	3.68

*SSD* Statistics Netherlands (own calculations)

^c^ Time-constant variable; all other variables are time-varying

**Table 5:** Logistic regression results for migration by separated and non-separated parents, presented as odds ratios relative to not migrating—control variables only

	Men				Women			
	OR	Z	95% CI		OR	Z	95% CI	
*Ties to own and partner’s parents (ref. none nearby or resident)*								
At least one parent resident	.91*	− 2.02	.82	1.00	.61***	− 8.63	.54	.68
Parent nearby	.24***	− 57.29	.23	.26	.25***	− 57.11	.24	.27
*At least one child of primary school age (4–12)*	.84***	− 5.26	.79	.90	.79***	− 7.03	.74	.84
*Age (ref.* < *30)*								
30–34	.90**	− 2.92	.85	.97	.91**	− 3.37	.86	.96
35–39	.74***	− 7.87	.69	.80	.71***	− 10.07	.66	.76
40–44	.59***	− 11.47	.54	.65	.49***	− 14.72	.44	.54
45 +	.46***	− 13.20	.41	.51	.39***	− 11.55	.34	.46
*Period 2009–2015 (ref. 2003–2008)*	.46***	− 20.64	.43	.50	.51***	.00	.47	.55
*Living in municipality of birth*	.51***	− 19.06	.48	.55	.45***	− 21.63	.42	.48
*International migrant status (ref. native Dutch) * ^c^								
First-generation immigrant	.66***	− 10.61	.61	.71	.62***	− 12.92	.58	.67
Second-generation immigrant	.97	− .72	.90	1.05	1.02	.41	.94	1.10
*Housing tenure (ref. owner-occupied)*								
Private rental	3.40***	24.61	3.08	3.75	2.72***	19.30	2.46	3.01
Social rental	.80***	− 4.75	.73	.88	.72***	− 7.94	.67	.78
Non-specific rental	2.25***	29.66	2.13	2.37	2.21***	29.69	2.10	2.33
Unknown housing tenure	1.57***	6.33	1.37	1.81	1.59***	6.48	1.38	1.82
*Completed education (ref. intermediate vocational training or lower)*								
Undergraduate degree	1.41***	11.61	1.33	1.49	1.36***	11.05	1.29	1.43
Graduate degree	2.00***	21.82	1.88	2.13	1.81***	19.15	1.70	1.92
*Employment status (ref. employed)*								
Self-employed	.87***	− 4.29	.82	.93	1.24***	5.59	1.15	1.34
Student	1.62***	3.80	1.26	2.08	1.93***	7.41	1.62	2.29
Not employed	1.39***	8.97	1.29	1.49	2.09***	29.13	1.99	2.19
*Equivalised household income, deciles (ref. 90–100%)*								
Income unknown	1.98***	5.67	1.57	2.52	1.51**	3.51	1.20	1.90
0–10%	1.14*	2.35	1.02	1.26	.90*	− 2.20	.81	.99
10–20%	.99	− .25	.89	1.10	.78***	− 4.92	.70	.86
20–30%	.89*	− 2.26	.81	.98	.79***	− 4.85	.72	.87
30–40%	.90*	− 2.34	.82	.98	.79***	− 4.96	.72	.87
40–50%	.77***	− 5.70	.71	.84	.73***	− 7.07	.67	.79
50–60%	.77***	− 6.02	.71	.84	.75***	− 6.58	.69	.82
60–70%	.85***	− 4.01	.79	.92	.82***	− 4.83	.75	.89
70–80%	.90**	− 2.73	.83	.97	.93†	− 1.91	.86	1.00
80–90%	.91*	− 2.42	.85	.98	.95	− 1.20	.88	1.03
*Urbanity of municipality (ref. not urban)*								
Hardly urban	1.14**	2.71	1.04	1.25	1.15**	2.97	1.05	1.26
Moderately urban	1.41***	7.09	1.28	1.55	1.42***	7.33	1.29	1.55
Strongly urban	1.65***	11.02	1.51	1.81	1.74***	12.47	1.60	1.90
Very strongly urban	1.75***	11.33	1.59	1.93	1.90***	13.31	1.73	2.09
*Jobs* < *50 km of municipality (ref.* < *1/2 million)*								
1/2–1 million	.84***	− 4.14	.78	.91	.81***	− 5.19	.74	.88
1–2 million	.85***	− 3.67	.77	.93	.84***	− 3.90	.77	.92
2 + million	.89†	− 1.90	.78	1.00	.88*	− 2.03	.78	1.00
*Avg housing costs per m2 in municipality (ref.* < *2000)*								
2000–2250	.94†	− 1.74	.87	1.01	.95	− 1.30	.89	1.02
2250–2500	.99	− .17	.90	1.09	.97	− .56	.89	1.07
2500 +	.80***	− 3.83	.72	.90	.80***	− 3.95	.72	.90
*Constant*	.01***	− 71.03	.01	.01	.01***	− 79.56	.01	.01

*SSD* Statistics Netherlands (own calculations)

Results for key predicting variables are shown in Table 2

^c^ Time-constant variable; all other variables are time-varying

*OR* odds ratio; *Z* Z statistic for testing OR = 1

^†^*p* < .10; **p* < .05; ***p* < .01; ****p* < .001

**Table 6:** Logistic regression results for migration after separation, presented as odds ratios relative to not migrating – control variables only

	Men				Women			
	OR	Z	95% CI		OR	Z	95% CI	
*Ties to own and partner’s parents (ref. none nearby or resident)*								
At least one parent resident	1.65***	5.52	1.38	1.98	.99	− .07	.77	1.28
Parent nearby	.47***	− 10.08	.40	.54	.49***	− 10.60	.43	.56
*At least one child of primary school age (4–12)*	1.17	1.36	.93	1.46	1.08	.68	.86	1.36
*Age (ref.* < *30)*								
30–34	.92	− .75	.73	1.15	.96	− .43	.81	1.15
35–39	.97	− .25	.76	1.23	.82†	− 1.91	.68	1.00
40–44	.80	− 1.64	.62	1.04	.68**	− 3.19	.54	.86
45 +	.77†	− 1.67	.57	1.05	.60**	− 2.79	.42	.86
*Period 2009–2015 (ref. 2003–2008)*	.76**	− 2.73	.62	.92	1.06	.61	.87	1.30
*Living in municipality of birth*	.75**	− 3.46	.64	.88	.73***	− 3.86	.62	.86
*International migrant status (ref. native Dutch) * ^c^								
First-generation immigrant	.87	− 1.23	.70	1.09	.99	− .08	.81	1.21
Second-generation immigrant	1.00	− .01	.80	1.25	1.20†	1.80	.98	1.46
*Housing tenure (ref. owner-occupied)*								
Private rental	2.21***	8.50	1.84	2.66	1.70***	5.17	1.39	2.07
Social rental	1.07	.74	.90	1.27	1.11	1.16	.93	1.32
Non-specific rental	1.83***	5.67	1.49	2.26	2.76***	9.86	2.26	3.38
Unknown housing tenure	1.83***	3.77	1.34	2.50	1.54*	2.07	1.02	2.33
*Completed education (ref. intermediate vocational training or lower)*								
Undergraduate degree	1.23*	2.13	1.02	1.49	1.12	1.23	.93	1.35
Graduate degree	1.28†	1.75	.97	1.68	1.46**	2.93	1.13	1.88
*Employment status (ref. employed)*								
Self-employed	.73**	− 3.00	.60	.90	1.03	.23	.79	1.34
Student	1.22	.39	.44	3.39	1.95**	2.72	1.21	3.15
Not employed	1.47***	4.38	1.24	1.74	1.72***	7.60	1.50	1.98
*Equivalised household income, deciles (ref. 90–100%)*								
Income unknown	.45*	− 2.01	.21	.98	.41*	− 1.97	.17	.99
0–10%	.83	− 1.26	.63	1.11	.94	− .31	.62	1.41
10–20%	.87	− .94	.64	1.17	.90	− .49	.60	1.36
20–30%	.77†	− 1.75	.58	1.03	.86	− .68	.57	1.31
30–40%	.83	− 1.37	.64	1.08	.87	− .63	.57	1.33
40–50%	.69**	− 2.75	.53	.90	.87	− .62	.57	1.34
50–60%	.74*	− 2.51	.58	.94	.85	− .72	.55	1.32
60–70%	.84	− 1.52	.66	1.05	.92	− .38	.59	1.43
70–80%	.58***	− 4.26	.46	.75	.82	− .84	.51	1.31
80–90%	.73**	− 2.72	.58	.92	1.13	.50	.70	1.82
*Urbanity of municipality (ref. not urban)*								
Hardly urban	1.06	.49	.83	1.35	1.01	.09	.80	1.27
Moderately urban	1.15	1.06	.89	1.48	.94	− .50	.73	1.20
Strongly urban	1.14	1.12	.90	1.45	1.12	.99	.89	1.40
Very strongly urban	.88	− .88	.67	1.17	.92	− .61	.71	1.20
*Jobs* < *50 km of municipality (ref.* < *1/2 million)*								
1/2–1 million	1.05	.43	.85	1.30	.99	− .05	.81	1.22
1–2 million	.80†	− 2.73	.62	1.04	.92	.61	.87	1.17
2 + million	.71†	− 1.83	.49	1.02	.76	− 1.54	.54	1.08
*Avg housing costs per m2 in municipality (ref.* < *2000)*								
2000–2250	.88	− 1.33	.72	1.06	.78**	− 2.55	.64	.94
2250–2500	1.08	.56	.82	1.42	.82	− 1.44	.63	1.07
2500 +	.91	− .58	.66	1.25	.85	− 1.04	.63	1.15
*Ex-partner in new union*	1.11	1.32	.95	1.28	.90	− 1.49	.78	1.03
*Years since separation*	.95**	− 2.94	.92	.98	.98	− 1.02	.95	1.01
*Moved out at separation * ^c^	1.37***	4.40	1.19	1.57	.97	− .49	.85	1.10
*Constant*	.01***	− 19.66	.01	.01	.01***	− 19.82	.00	.01

*SSD* Statistics Netherlands (own calculations)

Results for key predicting variables are shown in Table 3

^c^ Time-constant variable; all other variables are time-varying

OR: odds ratio; Z: Z statistic for testing OR = 1

^†^*p* < .10; **p* < .05; ***p* < .01; ****p* < .001

## Footnotes

1 Throughout this paper, the term ‘migration’ refers to internal migration.

2 See Thomson (2014) for a review of processes producing family complexity and Vidal and Huinink (2019) for a discussion on the interplay between spatial mobility and family dynamics.

3 The term ‘single parents’ is used to refer to separated parents who have not repartnered, thus excluding those who became parents whilst single. Furthermore, these are parents who are not in a co-residential partnership, regardless of whether their children live with them or not.

4 The term local ties is used as a synonym for location-specific capital (DaVanzo, 1981).

5 In shared residence arrangements (also called joint physical custody), children spend equal or near-equal time at both parents’ homes. We cannot identify these arrangements in our data as children can only be registered at one address. See Sect. 3.2 for more information about this data limitation.

6 Joint children with the new partner.

7 In 2009, the Promotion of Continued Parenting and Proper Divorce Act (Staatsblad, 2008) came into force, which promotes equal parenting responsibilities after divorce.

8 Following Van Ham et al. (2001), we use an absolute measure of the number of jobs in the municipality as an indicator of the total opportunity set rather than a relative measure such as the unemployment rate or the number of vacancies.

9 Of the anchors for whom we observe migration events during the window of observation, 11% have more than one migration event and 1% have more than two. The maximum number of observed migration events is five.
